# A Repolarização Precoce Afeta o Risco de Morte e Arritmias Ventriculares?

**DOI:** 10.36660/abc.20250275

**Published:** 2025-05-28

**Authors:** Guilherme Fenelon

**Affiliations:** 1 Hospital Israelita Albert Einstein São Paulo SP Brasil Hospital Israelita Albert Einstein, São Paulo, SP – Brasil

**Keywords:** Morte Súbita Cardíaca, Arritmias Cardíacas, Eletrocardiografia

O padrão de repolarização precoce (RP), definido como elevação do ponto J ≥1 mm em ≥2 derivações eletrocardiográficas (ECG) contíguas das regiões inferior e/ou lateral, é um achado comum, com prevalência de aproximadamente 6% na população adulta.^1,2^ A RP foi considerada por muito tempo um fenômeno benigno, uma vez que a maioria dos indivíduos afetados — geralmente homens jovens e atletas — apresenta uma incidência muito baixa de morte súbita cardíaca.^1,2^

No entanto, um estudo fundamental realizado por Haïssaguerre et al.^3^ demonstrou uma ocorrência significativamente maior de RP (31%) em pacientes com fibrilação ventricular (FV) idiopática em comparação aos controles (5%). Estudos subsequentes confirmaram a associação entre RP e arritmias ventriculares (AV) malignas em pacientes sem cardiopatia estrutural.^4,5^ Além disso, a RP tem sido associada a desfechos adversos em pacientes com distúrbios elétricos primários, como a síndrome do QT longo e a síndrome de Brugada.^2,6^

Ao contrário desses distúrbios, que são diagnosticados com base em anormalidades específicas no ECG, a síndrome de RP é identificada em pacientes reanimados de taquicardia ventricular polimórfica ou FV que apresentam padrões de RP.^1,2^ A maioria dos indivíduos com RP é assintomática, tornando a identificação de marcadores para estratificação de risco um aspecto crucial. As características de alto risco propostas incluem ondas J ≥2 mm, alterações dinâmicas na elevação do ponto J (>0,1 mV) e ondas J associadas a um segmento ST horizontal ou descendente.^1,2^ No entanto, o valor preditivo desses marcadores permanece incerto, e a RP transitória — presente no ECG basal, mas ausente em registros subsequentes — parece ser benigna.^7^

Os mecanismos da RP são pouco compreendidos. Estudos em animais sugerem que um aumento da corrente transitória de saída de potássio (I_to_) durante a fase 2 do potencial de ação desempenha um papel central ao promover a repolarização miocárdica prematura e a perda variável do platô do potencial de ação.^1,2,8^ Esse fenômeno é mais pronunciado no epicárdio, levando a uma heterogeneidade da repolarização, que subsequentemente favorece a reentrada de fase 2. A RP parece apresentar algum grau de hereditariedade, sendo mais prevalente em famílias de sobreviventes de parada cardíaca inexplicada.^1,2^ No entanto, os testes genéticos atualmente disponíveis para RP têm valor diagnóstico limitado.^2,8^

Pacientes diagnosticados com a síndrome de RP — aqueles reanimados após FV — necessitam de um cardiodesfibrilador implantável (CDI) para prevenção secundária.^1,2^ Episódios recorrentes de AV ocorrem em aproximadamente 40% dos casos, às vezes se manifestando como tempestades elétricas que exigem múltiplas descargas do CDI. A infusão de isoproterenol é eficaz para o manejo agudo, enquanto a quinidina (um bloqueador da corrente I_to_) pode reduzir as recorrências.^1,2,9^ Em casos selecionados, pode-se considerar a ablação por cateter direcionada aos focos de FV no trato de saída do ventrículo direito ou no sistema de Purkinje.^10^

O manejo de pacientes com padrões de RP é desafiador, uma vez que faltam marcadores de risco confiáveis para AV malignas e o prognóstico geral dessa população é muito favorável. Em indivíduos assintomáticos, recomenda-se apenas a orientação e o acompanhamento.^2,9^ Pode-se considerar o uso de um monitor de eventos implantável em pacientes com RP e síncope inexplicada.^2^

A prevalência da RP é influenciada por fatores como idade, sexo e etnia, sendo observada maior ocorrência entre afro-americanos.^1,9^ Consequentemente, sua frequência pode variar entre diferentes países. O estudo de Baldisserotto et al.^11^ oferece contribuições valiosas sobre o significado prognóstico da RP na população brasileira.

Este estudo retrospectivo, de coorte e de centro único, analisou 478 pacientes com RP, estratificando-os de acordo com o tipo de RP (lateral, inferior e inferolateral). Os desfechos avaliados incluíram a ocorrência de AV sustentada e a mortalidade por todas as causas durante um período de seguimento de 10 anos. A probabilidade de sobrevivência e as funções de risco cumulativo foram avaliadas por meio da análise de Kaplan-Meier, e modelos de regressão de Cox foram utilizados para ajuste de fatores de confusão, como idade e sexo.

A população do estudo era predominantemente composta por homens (74%), com idade média de 45,6 anos. Entre os participantes, 2,7% (13 pacientes) apresentaram AV e 2,3% (11 pacientes) morreram por todas as causas. Foram observadas diferenças significativas em relação à idade, sexo e prevalência de insuficiência cardíaca com fração de ejeção reduzida entre os subgrupos de RP. No entanto, a análise de Kaplan-Meier não revelou diferenças estatisticamente significativas nas taxas de sobrevivência ou de AV entre os tipos de RP (p=0,7 e p=0,5, respectivamente). Por outro lado, as estimativas de risco cumulativo variaram, com o grupo com RP inferolateral apresentando o maior risco de mortalidade.

Modelos de regressão de Cox ajustados não indicaram razões de risco significativas para AV ou mortalidade entre os diferentes tipos de RP, sendo a idade o único preditor significativo para ambos os desfechos. O estudo concluiu que a RP não impacta o risco de AV nem a mortalidade geral nessa coorte.

Embora o estudo forneça informações epidemiológicas valiosas, seu delineamento retrospectivo introduz potenciais vieses. Apenas um ECG foi analisado, o que impediu a avaliação de mudanças temporais no padrão de RP. O número relativamente pequeno de desfechos (AV e mortalidade) limita o poder estatístico para detectar diferenças entre os tipos de RP. Ainda assim, os achados são consistentes com relatos anteriores,^12^ sugerindo que o papel da RP como preditor de morte cardiovascular permanece incerto após o ajuste para fatores de confusão como idade, sexo e cardiopatia.

Em resumo, para a maioria dos indivíduos, a RP é um achado benigno que não requer intervenção além da orientação e do acompanhamento.^2,9^ Pacientes com RP que apresentem síncope inexplicada, histórico familiar forte de morte súbita cardíaca ou características eletrocardiográficas de alto risco necessitam de avaliação cuidadosa.^2^ São necessárias mais pesquisas para aprimorar a estratificação de risco em indivíduos assintomáticos que possam apresentar maior risco arrítmico.
