# Supplementary material for: A Repolarização Precoce Afeta o Risco de Morte e Arritmias Ventriculares?
Source: Arq Bras Cardiol. 2025 May 28;122(4):e20250275. [Article in Portuguese] doi: 10.36660/abc.20250275 (PMC12212379; doi:10.36660/abc.20250275)
Supplement: Translation

# Translation: Does Early Repolarization Affect the Risk of Death and Ventricular Arrhythmias?

**DOI:** 10.36660/abc.20250275i

**Authors:** Guilherme Fenelon

**Affiliations:** 1 São Paulo SP Brazil Hospital Israelita Albert Einstein, São Paulo, SP – Brazil

**Keywords:** Cardiac Arrhythmias, Cardiac Sudden Death, Electrocardiography

Early repolarization (ER) pattern, defined as J-point elevation of ≥1 mm in ≥2 contiguous inferior and/or lateral electrocardiography (ECG) leads, is a common finding, with a prevalence of approximately 6% in the adult population.^1,2^ ER has long been considered a benign phenomenon, as most affected individuals — typically young males and athletes — have a very low incidence of sudden cardiac death.^1,2^

However, a pivotal study by Haïssaguerre et al.^3^ demonstrated a significantly higher occurrence of ER (31%) in patients with idiopathic ventricular fibrillation (VF) compared to controls (5%). Subsequent studies confirmed an association between ER and malignant ventricular arrhythmias (VA) in patients without structural heart disease.^4,5^ Additionally, ER has been linked to adverse outcomes in patients with primary electrical disorders such as long QT syndrome and Brugada syndrome.^2,6^

Unlike these disorders, which are diagnosed based on specific ECG abnormalities, ER syndrome is identified in patients resuscitated from polymorphic ventricular tachycardia or VF who exhibit ER patterns.^1,2^ Most individuals with ER are asymptomatic, making the identification of markers for risk stratification crucial. Proposed high-risk features include J-waves ≥2 mm, dynamic changes in J-point elevation (>0.1 mV), and J-waves associated with a horizontal or descending ST segment.^1,2^ However, the predictive value of these markers remains uncertain, and transient ER — present on baseline ECG but absent on subsequent recordings — appears to be benign.^7^

The mechanisms of ER are poorly understood. Animal studies suggest that an increased transient outward potassium current (I_to_) during phase 2 of the action potential plays a central role by promoting premature myocardial repolarization and variable loss of the action potential dome.^1,2,8^ This phenomenon is more prominent in the epicardium, leading to repolarization heterogeneity, which subsequently promotes phase 2 reentry. ER appears to exhibit some degree of heritability, being overrepresented in families of unexplained cardiac arrest survivors.^1,2^ Nevertheless, current genetic testing for ER provides limited diagnostic value.^2,8^

Patients diagnosed with ER syndrome — those resuscitated from VF — require an implantable cardioverter-defibrillator (ICD) for secondary prevention.^1,2^ Recurrent VA episodes occur in approximately 40% of cases, sometimes presenting as electrical storms that necessitate multiple ICD discharges. Isoproterenol infusion is effective for acute management, while quinidine (an I_to_ blocker) may reduce recurrences.^1,2,9^ In selected cases, catheter ablation targeting VF triggers at the right ventricular outflow tract or the Purkinje system may be attempted.^10^

Managing patients with ER patterns is challenging, as reliable risk markers for malignant VA are lacking and the overall prognosis in this population is very favorable. In asymptomatic individuals, reassurance alone is recommended.^2,9^ An implantable loop recorder may be considered in patients with ER and unexplained syncope.^2^

The prevalence of ER is influenced by factors such as age, sex, and ethnicity, with a higher occurrence observed in African Americans.^1,9^ Consequently, its frequency may vary across different countries. The study by Baldisserotto et al.^11^ provides valuable insights into the prognostic significance of ER in the Brazilian population.

This retrospective, single-center cohort study analyzed 478 patients with ER, stratifying them by ER type (lateral, inferior, and inferolateral). Outcomes included sustained VA and all-cause mortality over a 10-year follow-up period. Survival probability and cumulative risk functions were assessed using Kaplan-Meier analysis, and Cox regression models were used to adjust for confounders such as age and sex.

The study population was predominantly male (74%), with a mean age of 45.6 years. Among the cohort, 2.7% (13 patients) experienced VA, and 2.3% (11 patients) died from all causes. Significant differences in age, sex, and the prevalence of heart failure with reduced ejection fraction were observed among ER subgroups. Nevertheless, Kaplan-Meier analysis showed no statistically significant differences in survival or VA rates across ER types (p=0.7 and p=0.5, respectively). However, cumulative risk estimates varied, with the inferolateral ER group demonstrating the highest mortality risk.

Adjusted Cox regression models indicated no significant hazard ratios for VA or mortality across ER types, with age emerging as the only significant predictor for both outcomes. The study concluded that ER does not impact the risk of VA or overall mortality in this cohort.

While the study provides valuable epidemiological insights, its retrospective design introduces potential biases. Only a single ECG was analyzed, preventing assessment of temporal changes in the ER pattern. The relatively small number of outcomes (VA and mortality) limits the statistical power to detect differences among ER types. Nevertheless, the findings are consistent with previous reports,^12^ suggesting that the role of ER as a predictor of cardiovascular death remains uncertain after adjusting for confounding factors such as age, sex, and heart disease.

In summary, for most individuals, ER is a benign finding that does not require intervention beyond reassurance.^2,9^ Patients with ER who present with unexplained syncope, a strong family history of sudden cardiac death, or high-risk ECG features require careful evaluation.^2^ Further research is needed to improve risk stratification in asymptomatic individuals who may be at increased arrhythmic risk.
